# The mediating role of E-health literacy and physical literacy in developing nutrition, physical activity, and health beliefs

**DOI:** 10.3389/fpubh.2026.1813151

**Published:** 2026-05-08

**Authors:** Mehmet Akarsu, Sinan Uğraş, Ahmet Enes Sağın, Mehmet Güllü, Taylan Akbuğa, Barış Mergan

**Affiliations:** 1 Faculty of Sport Sciences, İnönü University, Malatya, Türkiye; 2 Faculty of Sport Sciences, Çanakkale Onsekiz Mart University, Çanakkale, Türkiye; 3 Faculty of Sport Sciences, Bartın University, Bartın, Türkiye; 4 Department of Physical Education and Sports, Institute of Health Sciences, Inönü University, Malatya, Türkiye; 5 Faculty of Sports Sciences, Tokat Gaziosmanpaşa University, Tokat, Türkiye

**Keywords:** E-health literacy, health belief, healthy lifestyle belief, nutrition, physical activity, physical literacy

## Abstract

**Background:**

E-health literacy is considered an important factor that may influence adolescents’ ability to access, evaluate, and use digital health information, potentially shaping their health-related beliefs and behaviors. However, the mechanisms underlying this relationship remain insufficiently understood. Physical literacy has been suggested to facilitate the translation of health information into meaningful beliefs and actions. Therefore, this study aims to examine the relationship between e-health literacy and nutrition beliefs, physical activity beliefs, and health beliefs among high school students, with a particular focus on the potential mediating role of physical literacy.

**Methods:**

This study was conducted using the relational survey model, one of the quantitative research methods. A total of 816 high school students participated in the study; 484 were male, and 332 were female. Pearson correlation analysis was applied to examine the relationships between e-health literacy, healthy lifestyle beliefs and physical literacy. In the study, e-health literacy was considered the independent variable, healthy lifestyle beliefs were the dependent variable, and physical literacy was the mediating variable. To evaluate the effect of the mediating variable, the bias-corrected analysis method with 5,000 bootstraps was used, and it was verified that the confidence intervals did not contain zero values.

**Results:**

E-health literacy was significantly associated with nutrition beliefs, physical activity beliefs, and health beliefs both directly and indirectly. It was determined that physical literacy mediated the relationships between e-health literacy and nutrition beliefs, physical activity beliefs and health beliefs. The shared variance explained by e-health literacy and physical literacy was 48% for nutrition, 57% for physical activity, and 52% for health beliefs. It was also detected that e-health literacy accounted for 22% of the variance in physical literacy.

**Conclusion:**

The study showed that there was a correlation between e-health literacy and healthy lifestyle beliefs. As the level of e-health literacy increases, healthy lifestyle beliefs are also promoted. On the other hand, the study’s result showed that there was a positive significant relationship between e-health literacy and physical literacy. As a result, it was determined that physical literacy has a mediating effect on the relationship between e-health literacy and nutrition, physical activity, and health beliefs.

## 1 Introduction

Healthy lifestyle belief is a determinant concept in the processes of adopting and maintaining health behaviors (1, 2). This belief is defined as a potent psychological structure affecting individuals’ attitudes and behaviors toward health and quality of life. In the literature, it has been extensively discussed that healthy lifestyle beliefs increase the capacity of individuals to realize their health goals and ensure the sustainability of this process (3, 4). These beliefs are typically manifested across three fundamental behavioral domains: nutrition, physical activity, and general health attitudes. Each of these domains represents a critical dimension of individuals’ daily health-related decisions (1). Healthy lifestyle beliefs can be understood as a multidimensional psychological structure that reflects individuals’ tendencies and orientations toward sustaining well-being across these three domains. The nutrition dimension relates to beliefs and attitudes toward healthy dietary behavior and self-regulated eating practices; the physical activity dimension concerns engagement in regular exercise; and the health beliefs dimension captures individuals’ perceptions about their responsibility in maintaining health and adopting preventive behaviors. This framing enables a more holistic understanding of how beliefs drive actionable health behaviors in daily life (2, 5). Within this holistic framework, healthy lifestyle beliefs may also be interpreted as socio-culturally embedded constructs that are shaped by shared norms, values, and everyday practices related to food, movement, and health. A more detailed examination of the underlying mechanisms that shape healthy lifestyle beliefs is considered essential for effectively guiding individuals’ health behaviors (6). Understanding the specific factors that contribute to the development of these beliefs is therefore crucial for designing impactful health interventions. In particular, adolescence represents a critical developmental period in which health beliefs and lifestyle behaviors are actively formed (7). As adolescents begin to make more autonomous decisions, their beliefs become increasingly shaped by social and digital environments (8). Given adolescents’ high level of engagement with technology and their growing reliance on online sources for health-related information, digital contexts now play a central role in shaping their health beliefs and decision-making processes (9–11).

However, current evidence indicates that many adolescents face difficulties in maintaining healthy lifestyle behaviors. Insufficient physical activity, unhealthy dietary patterns, and increased sedentary screen time are common, alongside rising levels of stress, anxiety, and depressive symptoms (12, 13). These challenges may hinder the development of consistent and informed healthy lifestyle beliefs, highlighting the importance of identifying the mechanisms that support adolescents’ health-related belief formation. In this regard, understanding how adolescents access and process health-related information becomes particularly important.

As digitalisation continues to reshape healthcare access pathways, e-health literacy emerges as a critical factor influencing individuals’ healthy lifestyle beliefs. Defined as the capacity to access, assess, and apply health information via digital platforms, e-health literacy supports not only informational empowerment but also the development of sustained health behaviors (14, 15). Individuals with high levels of e-health literacy are more likely to find accurate information and implement it in their health routines particularly in nutrition, physical activity, and health maintenance as these competencies enable them to navigate health information environments more effectively within their socio-cultural and economic contexts (16, 17). In this context, e-health literacy can be understood as a foundational cognitive resource that contributes to the development of nutrition, physical activity, and health beliefs by enabling adolescents to interpret and apply digital health information.

In addition, the impact of e-health literacy is not only limited to behavioral level, but may also have an impact on psychological well-being. For example, Akingbade et al. (12) revealed that individuals with high levels of e-health literacy had lower levels of depression and anxiety. Similarly, in their study conducted in China, Yang et al. (13) reported a negative relationship between e-health literacy and levels of depression, insomnia and post-traumatic stress. These results indicate that e-health literacy is an important determinant of psychological well-being. Huang et al. (17) showed that college students with higher e-health literacy levels exhibited healthier eating and exercise habits.

On the contrary, insufficient e-health literacy has been linked to adverse outcomes such as difficulties accessing care, increased hospitalisations, treatment non-compliance, and mismanagement of chronic conditions (18). These challenges are especially pronounced during adolescence, a period marked by increased vulnerability to physical inactivity, unhealthy dietary patterns, and rising mental health problems such as stress, anxiety, and depressive symptoms, while health-related beliefs and behavioral competencies are still developing and remain highly modifiable (19).

However, access to information alone may not be sufficient for the development of stable and meaningful health beliefs. In this context, physical literacy, encompassing motivation, physical competence, and applied knowledge, not only serves as a behavioral framework but also provides a holistic literacy structure that enables individuals to interpret, internalize, and meaningfully engage with health-related information. Therefore, the contribution of e-health literacy to healthy lifestyle beliefs depends not only on access to information but also on the capacity to cognitively and perceptually internalize this information through physical literacy. This mediating role requires literacy to be considered as an integrated structure that connects cognitive understanding with bodily and experiential meaning-making processes, rather than as a direct transformation into behavior. In particular, physical literacy may function as a mechanism that enables adolescents to transform health information obtained from digital environments into consistent, internalized, and personally meaningful health beliefs.

From a theoretical perspective, e-health literacy and physical literacy can be conceptualized as two complementary dimensions of overall health literacy; one reflecting digital-cognitive competencies and the other representing bodily and interpretive literacy capacity. This dual structure allows physical literacy to be modeled as a mediating variable that fills the gap between access to digital health information and the formation of structured health beliefs, explaining the process rather than directly producing behavioral outcomes.

Indeed, existing studies demonstrate that physical literacy supports healthy lifestyle beliefs and practices (20, 21). Individuals who integrate physical activity into their daily routines and possess high levels of physical literacy are better able to apply digital health information particularly in adopting nutrition and exercise habits aligned with positive health beliefs (22–24). Moreover, physical literacy plays a critical role in facilitating the internalization and interpretation of health-related information, which is a fundamental requirement for the development of stable and conscious health belief systems (25, 26). These findings suggest that physical literacy may strengthen the pathway through which e-health literacy contributes to the development of health-related beliefs.

Despite the growing body of literature on e-health literacy and health behaviors, several important gaps remain. Previous studies have largely focused on direct relationships, giving limited attention to the mechanisms that explain how digital competencies translate into structured health beliefs. Moreover, the mediating role of physical literacy in this relationship has not been sufficiently examined among adolescents. Addressing these gaps, the present study contributes to the literature by conceptualizing healthy lifestyle beliefs as a multidimensional construct (nutrition, physical activity, and health beliefs) and by testing a mediation model that integrates e-health literacy and physical literacy within a unified framework. Focusing on high school students, this study provides a theoretically grounded explanation of how digital health competencies are transformed into sustainable health beliefs during adolescence.

Accordingly, the primary aim of this study is to examine the relationship between e-health literacy and healthy lifestyle beliefs specifically nutrition beliefs, physical activity beliefs, and health beliefs among high school students, and to test the mediating role of physical literacy in these relationships. In line with its proposed contributions, this study aims not only to identify the relationships among variables but also to clarify the mechanisms through which e-health literacy shapes health-related belief systems. Furthermore, the study offers implications for both theory development and evidence-based intervention design.

## 2 Materials and methods

### 2.1 Research design

This study was conducted using the relational survey design, one of the quantitative research methods. A relational survey design is a research model that aims to determine the relationships between two or more variables (27). In the study, the relationship between high school students’ e-health literacy levels and healthy lifestyle beliefs was examined, and the mediating role of physical literacy in this relationship was also evaluated. In the study’s theoretical model, e-health literacy was included as an independent variable, nutrition beliefs, physical activity beliefs, and nutrition, physical activity, and health beliefs were included as dependent variables, and physical literacy was included as a mediating variable.

### 2.2 Population and sample

The required sample size was determined using *a priori* power analysis for multiple regression analysis, which forms the basis of the mediation model tested in this study. The calculation was conducted using G*Power version 3.1.9.7 (28). Based on Cohen’s (29) recommendations, a medium effect size (*f*^2^ = 0.15), a significance level of *α* = 0.05, and a statistical power of 0.95 were assumed. According to this analysis, the minimum required sample size was calculated as 690 participants. The sample comprised 816 high school students. Participants were recruited using a convenience sampling method from public high schools located in the central districts of Malatya province, Türkiye. The selection included schools that granted permission to participate in the study and students who voluntarily agreed to be part of the research. While convenience sampling facilitated efficient data collection from a large adolescent sample, it may limit the generalizability of findings. Nevertheless, efforts were made to enhance sample diversity by including schools from different socioeconomic backgrounds and ensuring variation in age and gender across participants.

### 2.3 Data collection tools

#### 2.3.1 The healthy lifestyle beliefs scale for adolescents

The Healthy Lifestyle Beliefs Scale for Adolescents was adapted into Turkish by Kudubeş and Bektaş (5). In the original study, the internal consistency (Cronbach’s Alpha) of the scale was reported as 0.94, and the CFA results indicated an acceptable model fit (*χ*^2^ = 203.45, df = 93, RMSEA = 0.05, CFI = 0.96, GFI = 0.93, SRMR = 0.04). The Turkish version was found to be valid and reliable in a sample of 843 adolescents and has a total of 16 items and three sub-dimensions: health beliefs, physical activity and nutrition (5). The health beliefs sub-dimension reflects adolescents’ perceived ability to manage personal and social challenges related to health (e.g., “I know how to deal with things in a healthy way that will not bother me”, “I believe that I can reach the goals that I set for myself”). The physical activity sub-dimension captures beliefs related to engagement in and perceived benefits of physical activity (e.g., “I believe that exercise and being active will help me to feel better about myself”, “I believe that being active is fun”). The nutrition sub-dimension assesses beliefs regarding healthy eating behaviors (e.g., “I am certain that I will make healthy food choices”, “I know that I can make healthy snack choices regularly”). The scale is prepared according to the 5-point Likert system and is answered as “1=Strongly Disagree, 5=Strongly Agree”. A minimum score of 16 and a maximum score of 80 is obtained from the whole scale. An increase in the score obtained from the scale indicates that adolescents’ belief in healthy living increases. In the present study, internal consistency coefficients (Cronbach’s alpha) were calculated as 0.805 for health beliefs, 0.788 for physical activity, and 0.803 for nutrition, with an overall alpha of 0.906. The Confirmatory Factor Analysis results for the Healthy Lifestyle Beliefs Scale for Adolescents (*χ*^2^ = 435.748/Df = 94, CFI = 0.937, TLI = 0.920, NFI = 0.921, IFI = 0.937, RMSEA = 0.067, SRMR = 0.037) show that the scale has acceptable fit values (30).

#### 2.3.2 E-health literacy scale in adolescent

The e-Health Literacy Scale in Adolescents used in this study is the Turkish adapted version of the original eHealth Literacy Scale (eHEALS), which was developed by Norman and Skinner (31). In the original development study, Norman and Skinner reported a Cronbach’s alpha of 0.88, and a unidimensional factor structure explaining 56% of the variance was confirmed using exploratory factor analysis; confirmatory factor analysis was not conducted in that study (30). The Turkish version was validated for adolescents by Coşkun and Bebiş (32), with psychometric evaluations showing sufficient reliability and validity. In the Turkish adaptation, Coşkun and Bebiş (32) reported a Cronbach’s alpha of 0.88 and confirmatory factor analysis results of *χ*^2^/df = 1.70, RMSEA = 0.061, CFI = 0.99, IFI = 0.99, GFI = 0.98, AGFI = 0.94, which indicate a good model fit. The scale consists of 8 items and was developed to determine traditional literacy, health-related literacy, information retrieval, scientific research, media literacy and computer literacy. The scale items are organized as 1 = Strongly Disagree, 2 = Disagree, 3 = Undecided, 4 = Agree, 5 = Strongly Agree. The lowest score is 8 points, and the highest is 40 points. A high score on the scale indicates a high level of e-health literacy. In the current study, the Cronbach’s Alpha coefficient of the scale was calculated as 0.87. The results of the Confirmatory Factor Analysis performed for the E-Health Literacy Scale on the current research sample (*χ*^2^ = 41.110, Df = 14, CFI = 0.991, TLI = 0.982, NFI = 0.986, IFI = 0.991, RMSEA = 0.049, SRMR = 0.023) show that the scale has acceptable fit values (30).

#### 2.3.3 The perceived physical literacy scale for adolescents

The measurement tool developed by Sum et al. (33) was adapted into Turkish by Yılmaz and Kabak (34). The measurement tool, which measures adolescents’ perceived physical literacy, includes the dimensions of sense of self and self-confidence (3 items), self-expression and communication with others (3 items), and knowledge and understanding (3 items). The measurement tool is graded on a 5-point Likert scale. The average score of the scale can take values between 1 and 5. As the mean scores obtained from the sub-dimensions and the overall scale increase, it is inferred that the perceived physical literacy is high. In the original study by Sum et al. (33), the scale showed good internal consistency with a Cronbach’s Alpha of 0.82. The CFA results also indicated acceptable model fit (*χ*^2^ = 49.74, df = 24, CFI = 0.97, RMSEA = 0.05). In the Turkish adaptation study by Yılmaz and Kabak (34), the scale showed a Cronbach’s Alpha of 0.84, and the CFA results were reported as *χ*^2^ = 66.28, df = 24, CFI = 0.96, RMSEA = 0.06, indicating acceptable fit. In the current study, Cronbach’s Alpha coefficient was calculated as 0.85 for the whole scale. The confirmatory factor analysis results (*χ*^2^ = 65.612/Df = 22, CFI = 0.984, TLI = 0.974, NFI = 0.976, IFI = 0.984, RMSEA = 0.049, SRMR = 0.024) for the physical literacy scale on the research sample show that the scale has acceptable fit values (30). Although the subscale names do not directly reference physical activity behavior, they reflect core components that influence such behavior, consistent with Whitehead’s Physical Literacy Framework. According to Whitehead (20), physical literacy is defined as “the motivation, confidence, physical competence, knowledge and understanding to value and take responsibility for engagement in physical activities for life.” In this framework, each sub-dimension sense of self, social interaction, and knowledge contributes to the cognitive, affective, and social foundations of physical activity behavior. For example, confidence and self-perception impact an adolescent’s willingness to participate in physical activity; communication and social skills affect their engagement in social physical contexts; and understanding of physical health principles informs sustainable behavior. Therefore, while not measuring behavior directly, the scale aligns theoretically with behavioral enablers.

### 2.4 Data collection procedure

Prior to data collection, participants were provided with detailed information regarding the purpose, scope, and confidentiality principles of the study. The data collection process was conducted entirely on a voluntary basis. Data were collected in classroom settings within public high schools under the direct supervision of researchers experienced in conducting field studies with adolescents. To minimize distraction and psychological burden, data collection was carried out during regular school hours and outside examination periods.

Before participation, all students were presented with an informed consent form clearly stating that participation was voluntary, that responses would be used solely for scientific purposes, and that all personal information would be kept confidential. Since the participants were under the age of 18, an information sheet explaining the purpose, scope, ethical principles, and confidentiality procedures of the study, along with a parental consent form, was delivered to parents via school administration. Only students who obtained written parental consent were included in the study. In addition, informed assent was obtained from the students prior to data collection.

Participants were explicitly informed that there were no right or wrong answers and that responses should reflect their own honest and personal opinions. This approach was adopted to reduce social desirability bias. Data were collected using a structured data collection form developed in line with the objectives of the study. First, demographic information was obtained, followed by the administration of the measurement tools in a fixed order: the E-Health Literacy Scale for Adolescents, the Perceived Physical Literacy Scale for Adolescents, and the Healthy Lifestyle Beliefs Scale for Adolescents.

To ensure data completeness, participants were asked to respond to all items, and researchers provided guidance to prevent missing data. Particular attention was paid to maintaining participant motivation throughout the data collection process. The application took approximately 10–15 min. The administration environment was quiet, orderly, and free from distractions. Clear written and verbal instructions were provided to ensure that participants fully understood the process, to reduce fatigue, and to improve response accuracy.

All data were collected anonymously and were not associated with any personally identifiable information. The data obtained were used solely for the purposes of this study. The research process was conducted in accordance with ethical principles for studies involving human participants and complied with the Declaration of Helsinki.

### 2.5 Data analysis

Jasp 0.18.3.0 statistical program was used for data analysis. Skewness and kurtosis values were examined to evaluate the normality distribution of the data. Kline stated that the kurtosis and skewness values should be less than three for the data set to show a normal distribution (35). The results indicated that all variables had skewness and kurtosis values within the acceptable range (±3), confirming that the data were normally distributed. Pearson correlation analysis was applied to examine the relationships between e-health literacy, health beliefs, physical activity, nutrition, and physical literacy. In the study, e-health literacy was defined as the independent variable, nutrition, physical activity, and health beliefs as dependent variables, and physical literacy as the mediating variable. To examine the mediating role of physical literacy, a structural equation modeling approach was employed. The hypothesized model included both direct paths from e-health literacy to the dependent variables and indirect paths through physical literacy. Model estimation was conducted using maximum likelihood estimation. The significance of indirect effects was tested using a bias-corrected bootstrap procedure with 5,000 resamples. Mediation effects were considered statistically significant when the 95% confidence intervals did not include zero (36).

## 3 Results

As presented in Table 1, a total of 816 high school students participated in the study, including 484 males (59.3%) and 332 females (40.7%). In terms of grade distribution, 253 participants (31.0%) were in the 1st grade, 168 (20.6%) in the 2nd grade, 198 (24.3%) in the 3rd grade, and 197 (24.1%) in the 4th grade. Regarding physical activity status, 241 participants (29.5%) reported engaging in regular sports activities, whereas 575 (70.5%) did not. The mean age of the participants was 15.41 years (SD = 1.19), ranging from 13 to 19 years (median = 15.00). The average height was 166.58 cm (SD = 12.57), and the average weight was 56.74 kg (SD = 12.84). In addition, descriptive statistics for the main study variables indicated that the mean scores were 3.45 (SD = 0.88) for e-health literacy, 3.69 (SD = 0.79) for physical literacy, 3.53 (SD = 0.89) for nutrition, 3.75 (SD = 0.85) for physical activity, and 3.69 (SD = 0.80) for health beliefs.

**Table 1:** Descriptive characteristics of the participants.

Variable	Category/Statistic	*n*	%	Mean ±SD	Min–Max
Gender	Male	484	59.3	–	–
	Female	332	40.7	–	–
Grade level	1st grade	253	31.0	–	–
	2nd grade	168	20.6	–	–
	3rd grade	198	24.3	–	–
	4th grade	197	24.1	–	–
Physical activity status	Regular activity	241	29.5	–	–
	No regular activity	575	70.5	–	–
Age (years)	–	–	–	15.41 ± 1.19	13–19
Height (cm)	–	–	–	166.58 ± 12.57	–
Weight (kg)	–	–	–	56.74 ± 12.84	–
EHL	–	–	–	3.45 ± 0.88	–
PL	–	–	–	3.69 ± 0.79	–
N	–	–	–	3.53 ± 0.89	–
PA	–	–	–	3.75 ± 0.85	–
HB	–	–	–	3.69 ± 0.80	–

Values are presented as frequency (n), percentage (%), or mean ± standard deviation (SD). *n*, total number of participants. EHL, E-health literacy; PL, Physical literacy; N, Nutrition; PA, Physical activity; HB, Health belief.

As presented in Table 2, significant positive correlations were observed among all study variables. E-health literacy was positively correlated with physical literacy (*r* = 0.480, *p* < 0.001), nutrition (*r* = 0.468, *p* < 0.001), physical activity (*r* = 0.450, *p* < 0.001), and health beliefs (*r* = 0.441, *p* < 0.001). Physical literacy was also significantly associated with nutrition (*r* = 0.672, *p* < 0.001), physical activity (*r* = 0.747, *p* < 0.001), and health beliefs (*r* = 0.715, *p* < 0.001). These findings suggest that higher levels of e-health literacy and physical literacy are associated with more favorable healthy lifestyle belief patterns among adolescents.

**Table 2:** Pearson correlation coefficients between study variables.

Variables	EHL	PL	N	PA	HB
EHL	–	0.480	0.468	0.450	0.441
PL	–	–	0.672	0.747	0.715
N	–	–	–	0.695	0.683
PA	–	–	–	–	0.672
HB	–	–	–	–	–

All values represent Pearson correlation coefficients (r). All correlations are statistically significant (*p* < 0.001). EHL, E-health literacy; PL, Physical literacy; N, Nutrition; PA, Physical activity; HB, Health belief.

The findings regarding the mediating role of physical literacy (PL) in the relationship between e-health literacy (EHL) and healthy lifestyle beliefs are presented in Table 3. The results of the direct effects indicated that PL had significant positive effects on physical activity (PA) [*β* = 0.690, 95% CI (0.632, 0.744)], health beliefs (HB) [*β* = 0.654, 95% CI (0.590, 0.712)], and nutrition (N) [*β* = 0.581, 95% CI (0.518, 0.641)]. Similarly, EHL showed significant direct effects on PA [*β* = 0.137, 95% CI (0.071, 0.200)], HB [*β* = 0.146, 95% CI (0.073, 0.216)], and N [*β* = 0.216, 95% CI (0.144, 0.291)]. In addition, EHL had a strong and significant effect on PL [*β* = 0.546, 95% CI (0.455, 0.633)]. Regarding indirect effects, EHL had significant indirect effects on N [*β* = 0.317, 95% CI (0.267, 0.368)], PA [*β* = 0.377, 95% CI (0.322, 0.432)], and HB [*β* = 0.357, 95% CI (0.303, 0.411)] through PL, indicating a mediating role of physical literacy. The total effects of EHL on N [*β* = 0.533, 95% CI (0.464, 0.603)], PA [*β* = 0.513, 95% CI (0.444, 0.583)], and HB [*β* = 0.503, 95% CI (0.432, 0.573)] were also significant. Overall, these findings suggest that physical literacy plays a substantial mediating role in the relationship between e-health literacy and healthy lifestyle belief dimensions. The model explained 48% of the variance in nutrition, 57% in physical activity, and 52% in health beliefs, while e-health literacy explained 22% of the variance in physical literacy.

**Table 3:** Structural equation modeling results of direct, indirect, and total effects among study variables (*n* = 816).

Direct effects			*β*	Std. Error	%95 Confidence interval		
					*p*	Lower	Upper
PL	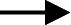	PA	0.690	0.026	<0.001	0.632	0.744
EHL	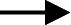	PA	0.137	0.030	<0.001	0.071	0.200
PL	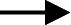	HB	0.654	0.028	<0.001	0.590	0.712
EHL	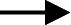	HB	0.146	0.031	<0.001	0.073	0.216
PL	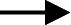	N	0.581	0.029	<0.001	0.518	0.641
EHL	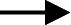	N	0.216	0.033	<0.001	0.144	0.291
EHL	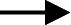	PL	0.546	0.035	<0.001	0.455	0.633

Indirect effects					*β*	Std. Error	%95 Confidence interval		
							*p*	Lower	Upper
EHL	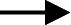	PL	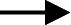	N	0.317	0.026	<0.001	0.267	0.368
EHL	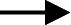	PL	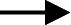	PA	0.377	0.028	<0.001	0.322	0.432
EHL	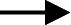	PL	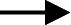	HB	0.357	0.027	<0.001	0.303	0.411

Total effects			*β*	Std. Error	%95 Confidence interval		
					*p*	Lower	Upper
EHL	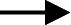	N	0.533	0.035	<0.001	0.464	0.603
EHL	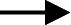	PA	0.513	0.036	<0.001	0.444	0.583
EHL	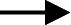	HB	0.503	0.036	<0.001	0.432	0.573

The variance explained	*R* ^2^
N	0.478
PA	0.570
HB	0.524
PL	0.229

Results are based on structural equation modeling. Indirect effects were tested using a bias-corrected bootstrap procedure with 5,000 resamples. Mediation is considered significant when the 95% confidence interval does not include zero. *R*^2^: The variance explained. EHL, E-health literacy; PL, Physical literacy; N, Nutrition; PA, Physical activity; HB, Health belief.

**Figure 1:**
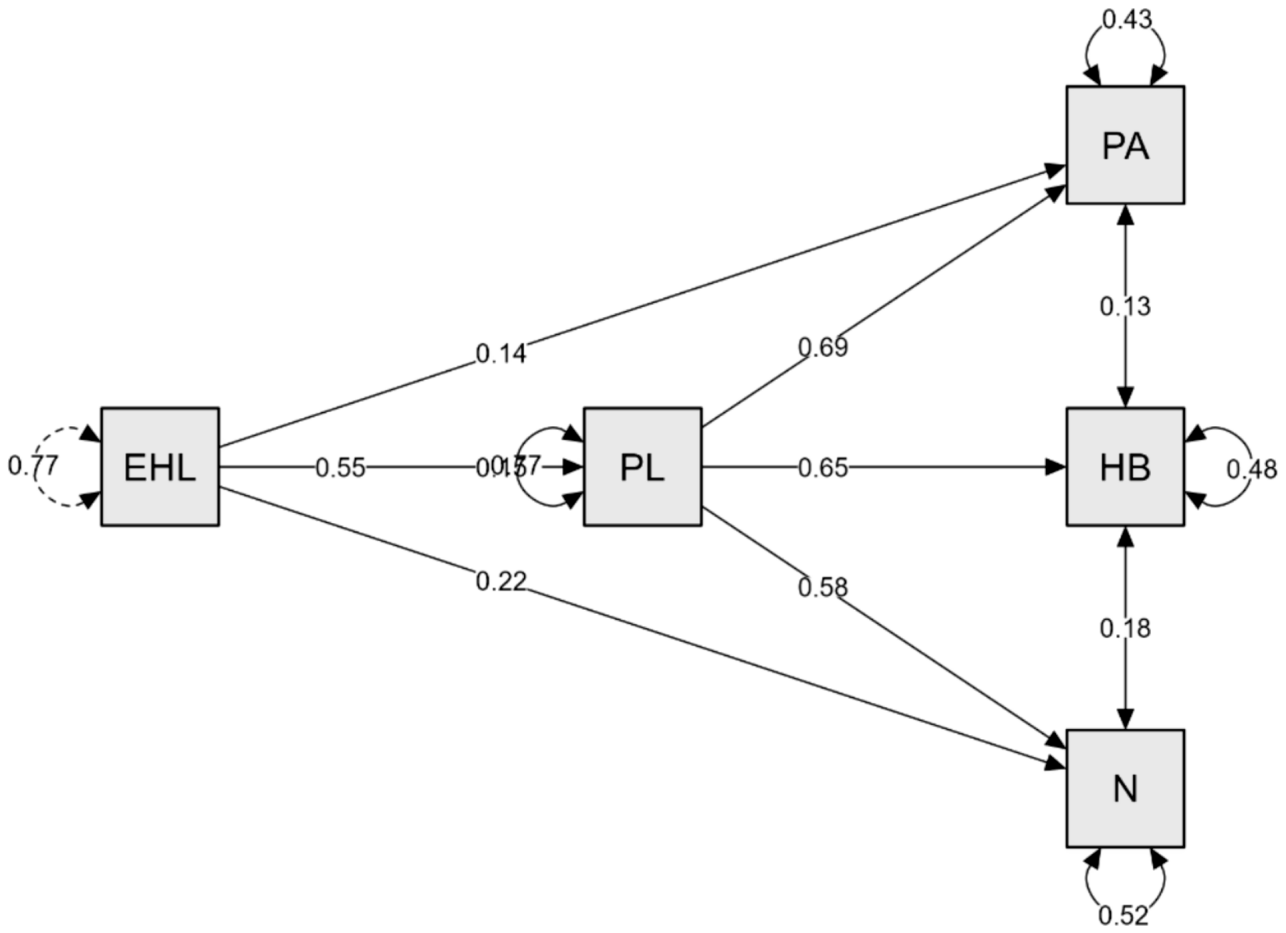
Structural equation model of the mediating role of physical literacy in the relationship between e-health literacy and healthy lifestyle belief dimensions among high school students (*N* = 816). Standardized path coefficients (*β*) are presented.

Figure 1 presents the structural equation model illustrating the mediating role of PL in the relationship between EHL and healthy lifestyle belief dimensions. The results indicate that EHL positively predicts PL (*β* = 0.55). In turn, PL significantly predicts PA (*β* = 0.69), HB (*β* = 0.65), and N (*β* = 0.58). In addition to these indirect pathways, EHL also demonstrates direct effects on PA (*β* = 0.14), HB (*β* = 0.15), and N (*β* = 0.22).

## 4 Discussion

The aim of this study was to test the mediating role of physical literacy in the relationship between e-health literacy and healthy lifestyle beliefs in high school students. Overall, the findings revealed that e-health literacy was positively associated with nutrition, physical activity, and health beliefs, both directly and indirectly through physical literacy. In addition, physical literacy demonstrated a significant mediating role across all three dimensions, suggesting that it may function as a key mechanism linking digital health competencies to structured health-related beliefs. Additionally, e-health literacy showed significant direct relationships with all healthy lifestyle belief dimensions, while also significantly predicting physical literacy. Physical literacy, in turn, was strongly associated with nutrition, physical activity, and health beliefs, and mediated the relationships between e-health literacy and these outcomes.

Specifically, this study examined whether physical literacy mediates the relationship between e-health literacy and nutrition beliefs, physical activity beliefs, and health beliefs. According to the findings, e-health literacy has a direct, positive and significant effect on nutrition. According to the findings, e-health literacy showed a direct, positive and significant relationship with nutrition. Studies reveal that individuals with high e-health literacy are more skilled in adapting this information to their dietary habits by accessing accurate and reliable information (14, 37), and this aligns with evidence indicating that higher e-health literacy is associated with healthier eating patterns and improved dietary decision-making among adolescents and young adults (14, 38, 39). This is due to the fact that individuals increase their ability to make conscious and healthy decisions by receiving personalized nutritional recommendations from digital environments (40, 41). In addition, it is observed that individuals with high e-health literacy make more informed dietary choices thanks to their ability to distinguish accurate and reliable sources from information pollution in digital environments (42, 43). While previous studies have largely focused on behavioral outcomes such as dietary practices, the present findings indicate that e-health literacy is associated with the formation of nutrition-related beliefs rather than behavior itself. Thus, e-health literacy appears to be linked to how adolescents perceive, interpret, and cognitively evaluate nutrition-related information.

E-health literacy was found to be positively associated with physical activity. Studies have reported that high e-health literacy is positively associated with increased physical activity levels (44) and that individuals with high e-health literacy are more likely to participate in physical activity (45). Systematic reviews reveal that these individuals not only increase the likelihood of participation in physical activity but also have a better understanding of the health benefits of these activities (46). This may be explained by the fact that individuals with high e-health literacy develop a stronger cognitive understanding of physical activity as an important component of a healthy lifestyle through digital information sources. In particular, the ability to access, interpret, and evaluate physical activity-related information may shape individuals’ perceptions and beliefs about physical activity (47, 48). Accordingly, the findings suggest that e-health literacy is associated with physical activity beliefs that give meaning to the concept of being physically active, rather than directly reflecting behavioral engagement.

When considered in the context of health beliefs, e-health literacy is positively associated with individuals’ adoption of health-related behaviors (46). It plays a significant role, especially in critical issues such as treatment adherence and patient satisfaction (49). In addition, studies have revealed that information-seeking and sharing behaviors on social media improve individuals’ health beliefs along with e-health literacy (50). Therefore, this result can be explained by the fact that high school students can easily access health-related information thanks to their predisposition to digital platforms (51), gain the ability to understand and evaluate this information, and are exposed to content that supports health behaviors through platforms such as social media (52). In line with prior research emphasizing the role of health literacy in shaping health-related decision-making and attitudes (18, 46, 49), these findings underscore that e-health literacy may function as an important cognitive factor associated with the development of adolescents’ health beliefs, particularly during a developmental stage where such beliefs are still forming (7, 19). These findings support the role of e-health literacy as a key cognitive determinant in the development of adolescents’ health beliefs.

The positive relationship between e-health literacy and nutrition appears to be strengthened through the mediating role of physical literacy. Research shows that physical literacy increases individuals’ nutritional awareness (52) and encourages healthy dietary habits (53). Therefore, individuals with high e-health literacy can better comprehend the balance between physical activity and nutrition by using the information they obtain through digital platforms and can integrate this information into their daily lives more effectively (37, 45). This is consistent with studies indicating that physical literacy is associated with healthier lifestyle patterns, including improved nutritional awareness and dietary quality (52–54). In this context, physical literacy may be considered a cognitive–perceptual construct that supports the development of structured nutrition beliefs. Accordingly, the present findings suggest that physical literacy may mediate the process through which digital health information is internalized and reflected in nutrition-related beliefs.

The relationship between e-health literacy and physical activity beliefs appears to be strengthened through the mediating role of physical literacy. Physical literacy is an important factor that enhances individuals’ ability to plan and organize physical activities and set health goals (55). Individuals with high e-health literacy may process physical activity-related information more effectively and integrate it into their cognitive frameworks with the support of physical literacy (56). The literature suggests that physical literacy contributes to individuals’ awareness and understanding of physical activity (57–59). In addition, physical literacy enables individuals to interpret physical activity not merely as a health goal but as a health behavior integrated into daily life (60). Within this framework, it can be argued that e-health literacy shapes physical activity beliefs among high school students through the mediating role of physical literacy. In other words, physical literacy may be considered a key mediating mechanism that explains the relationship between e-health literacy and the formation of physical activity beliefs.

The relationship between e-health literacy and health beliefs appears to be strengthened through the mediating role of physical literacy. Studies reveal that physical literacy enables individuals to understand health information better, helps them integrate it into their health behaviors (56) and increases their motivation to achieve their health goals by strengthening their health beliefs (61, 62). This situation reveals that individuals go beyond passively learning health-related information and actively participate in the processes of transforming this information into health behaviors. Especially for individuals with high e-health literacy, the effect of physical literacy becomes more evident since these individuals can easily access reliable health information through digital platforms and combine this information with disease prevention and health promotion strategies (63). The contribution of physical literacy extends beyond comprehension and includes strengthening individuals’ perceptions of self-efficacy (64, 65) and awareness of health-related risks (63). Accordingly, physical literacy may play a meaningful mediating role in shaping how e-health literacy is reflected in adolescents’ health beliefs.

## 5 Strengths and limitations

This study provides a theoretically grounded model examining the relationships between e-health literacy, physical literacy, and healthy lifestyle beliefs, thereby contributing to the existing literature. In particular, this study contributes by empirically demonstrating the mediating role of physical literacy in the relationship between e-health literacy and nutrition beliefs, physical activity beliefs, and health beliefs. However, the framework presented should be considered as a partial representation of the relationships among health literacy and health belief constructs, rather than a comprehensive model. Another important limitation of this study is the use of a convenience sampling method. This sampling approach may introduce selection bias, as participants were recruited from accessible schools and volunteered to participate, which may limit the representativeness of the sample. Although the sample size (*N* = 816) can be considered adequate for statistical analysis, it may not be sufficient to generalize the findings to broader populations. Therefore, the findings should be interpreted with caution in terms of external validity. In addition, since the participant group consisted only of high school students, the generalizability of the findings is limited to similar age groups and contexts. Furthermore, the cross-sectional design of the study restricts the ability to draw conclusions about the directionality of relationships among variables. Finally, the study focused on a limited set of variables, and other potential mediating or moderating factors were not included.

## 6 Conclusion

In conclusion, e-health literacy is an important variable associated with individuals’ healthy lifestyle beliefs. This study demonstrated that e-health literacy is positively and significantly related to nutrition, physical activity, and health beliefs, while physical literacy plays a critical mediating role in these relationships. Specifically, the mediation analysis revealed that physical literacy has a partial mediating role in the relationships between e-health literacy and each sub-dimension of healthy lifestyle beliefs. These findings indicate that e-health literacy is associated with the development of nutrition beliefs, physical activity beliefs, and health beliefs both directly and indirectly through physical literacy. Furthermore, higher levels of e-health literacy and physical literacy are associated with stronger healthy lifestyle beliefs. However, considering the cross-sectional nature of the study, future research should prioritize longitudinal and intervention-based designs to provide stronger evidence regarding the role of e-health literacy and physical literacy in shaping healthy lifestyle beliefs. In this context, there is a particular need for intervention studies examining the effectiveness of programs aimed at improving e-health literacy and physical literacy among adolescents. Such studies would provide a more robust basis for developing evidence-based educational and public health strategies. Accordingly, this study highlights the potential importance of these constructs and emphasizes the need for further research to inform the design of targeted interventions. Additionally, future research may explore reverse or bidirectional models in which health beliefs are considered antecedents of health literacy, particularly in adult or clinical populations. Moreover, replicating this study across different age groups and cultural contexts would contribute significantly to the literature by evaluating the generalizability of the findings.

## Data Availability

The datasets used and/or analysed during the present study are available from the corresponding author upon reasonable request.
